# Case Report: Bilateral diaphragmatic dysfunction due to *Borrelia Burgdorferi*

**DOI:** 10.12688/f1000research.5375.1

**Published:** 2014-10-06

**Authors:** Suhail Basunaid, Chris van der Grinten, Nicole Cobben, Astrid Otte, Roy Sprooten, Rohde Gernot

**Affiliations:** 1 Department of Respiratory Medicine, Maastricht University, Medical Centre, Maastricht, 6200 MD, Netherlands; 2 Centre of Home Mechanical Ventilation, Maastricht University, Medical Centre, Maastricht, 6200 MD, Netherlands

**Keywords:** Lyme disease, diaphram, Borrelia burgdorferi, hypoventilation

## Abstract

**Summary:**

In this case report we describe a rare case of bilateral diaphragmatic dysfunction due to Lyme disease.

**Case report:**

A 62-years-old male presented to the hospital because of flu-like symptoms. During initial evaluation a bilateral diaphragmatic weakness with orthopnea and nocturnal hypoventilation was observed, without a known aetiology. Bilateral diaphragmatic paralysis was confirmed by fluoroscopy with a positive sniff test. The patient was referred to our centre for chronic non-invasive nocturnal ventilation (cNPPV). Subsequent investigations revealed evidence of anti- *Borrelia* seroactivity in EIA-IgG and IgG-blot, suggesting a recent infection with Lyme disease, and resulted in a 4-week treatment with oral doxycycline. The symptoms of nocturnal hypoventilation were successfully improved with cNPPV. However, our patient still shows impaired diaphragmatic function but he is no longer fully dependent on nocturnal ventilatory support.

**Conclusion:**

Lyme disease should be considered in the differential diagnosis of diaphragmatic dysfunction. It is a tick-borne illness caused by one of the three pathogenic species of the spirochete *Borrelia burgdorferi*, present in Europe. A delay in recognizing the symptoms can negatively affect the success of treatment. Non-invasive mechanical ventilation (NIV) is considered a treatment option for patients with diaphragmatic paralysis.

## Introduction

Patients with bilateral diaphragmatic paralysis may initially present with dyspnea, orthopnea, and as the disease progresses respiratory failure. Bilateral diaphragmatic paralysis is a severe generalized muscle weakness, however in few cases it has been observed that the diaphragm can be the only involved organ. The most common causes of bilateral diaphragmatic paralysis are damage to the phrenic nerves and generalized muscle diseases. Nocturnal ventilatory assistance may have a significant beneficial effect ^6^. These patients show reduced ventilatory muscle strength, as measured by maximal inspiratory and trans-diaphragmatic pressures. These symptoms could improve in association with an improved functional score and decreased dyspnea under ventilatory assistance. Non-invasive positive pressure ventilation (NPPV) is the therapeutic tool of choice for symptomatic patients with bilateral diaphragmatic paralysis.

This case report describes the development of diaphragmatic paralysis in a patient with Lyme disease with the need for ventilatory support ^3, 4^.

Lyme disease is a tick-borne illness caused by the spirochete *Borrelia burgdorferi*. There are three species of the *Borrelia*, all of them appear in Europe, and two appear in Asia. Lyme disease has a broad spectrum of clinical manifestations and varies in severity. Regarding the clinical manifestations of Lyme disease, three phases have been described: early localized, early disseminated and late disease. Early localized disease is characterized by the appearance of the erythema migrans, with or without constitutional symptoms. The early-disseminated disease is characterized by multiple lesions, and the late disease is typically associated with intermittent or persistent arthritis involving one or a few large joints, especially the knee. Late Lyme disease may develop months to a few years after the initial infection ^2^.

## Case report

A 62-year-old male was referred to our hospital as a second opinion for further analysis of respiratory failure due to bilateral diaphragm dysfunction. He presented initially with flu-like symptoms. These consisted of low-grade fever, arthralgia in the neck and shoulders and symptoms of nocturnal hypoventilation. The symptoms started months before the actual clinical presentation and led to deterioration of the patient’s general condition.

Initially there was also a skin rash at the back of his right leg due to an unnoticed tick-bite. The rash started in the form of a ring, later progressed to a size of 10 cm in diameter. At that time the patient had also developed a numbness of the left-side of his face. This gradually resolved during the next days. He complained of dyspnoea that was worse on supine position. There was no evidence of motor/sensory abnormality in the extremities. He had no headache, but he was complaining of neck and shoulder stiffness. He developed a low-grade fever (38.7°C) without shivering. Gradually, fatigue and inactivity evolved.

The patient is an otherwise healthy Caucasian carpenter. He is married and has two healthy kids. He took no medication, had stopped smoking 32 years earlier and drank 2 units alcohol per day. History of allergy developed later when he started ceftriaxon as a second choice for peripheral neuroborreliosis. He works as a volunteer for a forest preservation fund. As a hobby he liked to walk in the woods and he was not aware of any tick-bite.

Initially on physical examination, the patient was hemodynamically stable and not febrile. The fundoscopic exam was normal. The neck was supple and there was no evidence of positive meningeal signs. On percussion the left lung base was higher situated than the right lung base. In upright position our patient had a breathing rate of 24 per minute and SpO2 of 97%. Lying down for 45 second caused severe shortness of breath and an increase in respiratory rate to 40 per minute. Paradoxical breathing was observed and the saturation dropped to 91%.

**Figure 1.**
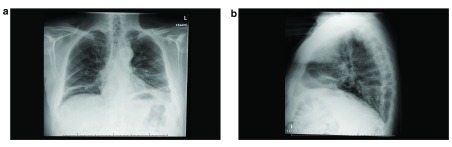
( **a**) Frontal chest radiograph during initial presentation. ( **b**) Lateral chest radiograph during initial presentation.

**Figure 2.**
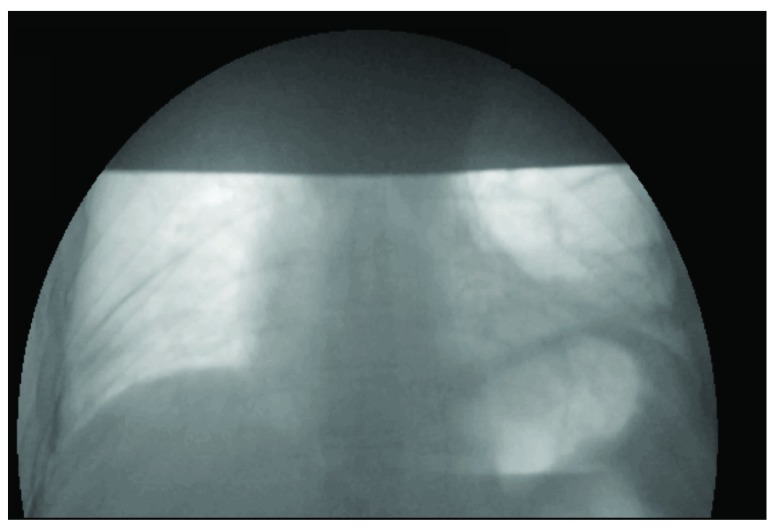
Fluoroscopy with sniff test.

The chest radiographs ( Figure 1a and 1b) demonstrated an elevated left hemi-diaphragm. Screening of diaphragmatic movement during fluoroscopy with sniff manoeuvres revealed a paradoxical movement of both hemi-diaphragms ( Figure 2). A pulmonary function test revealed a decrease in supine vital capacity of more than 20% of predicted ( Table 2). Arterial blood gases showed pH 7.40, PaCO _2_ 4.9kPa, PaO _2_ 7.8kPa, HCO _3_ 24.6 mmol/l, base excess -0.2 mmol/l. Antibodies to extractable nuclear antigens SSA, SSB, RNP, Sm, SCL-70, Jo-1 and serology of Q-fever were negative. IgG antibodies to *B. burgdorferi* were detectable in serum.

**Table 2.** Pulmonary function test.

Substance	Pred	Upright	% of pred. value	LLN	ULN	Supine	% of pred. value	% change
FEV1 (L)	3.37	1.79	53.2	75.2	124.8	0.53	15.9	29.8
FVC1IN (L)	4.47	1.84	41.2	79.5	120.5	0.58	12.9	31.5
FEV1%VCmax (%)	76.1	79.12	104.0	84.5	115.5	77.67	102.1	98.2
FVC (L)	4.30	2.26	52.6	76.7	123.3	0.69	16.0	30.4
PEF (L/s)	8.41	8.53	101.4	76.4	123.6	1.28	15.2	15.0
PIF (L/s)		5.38				1.34		24.8
FRC (L)	3.63	2.57	70.7	72.9	127.1			
RV (L)	2.47	2.32	94.1	72.7	127.3			
TLC (L)	7.14	4.80	67.1	83.9	116.1			
RV%TLC (%)	38.1	48.40	126.9	76.5	123.5			
FRC%TLC (%)	56.8	53.58	94.3	80.5	119.5			

FEV1: Forced expiratory volume in 1 second FVC: Forced vital capacity PEF: Expiratory peak flow PIF: Peak inspiratory flow FRC: Functional residual capacity RV: Residual volume TLC: Total lung capacity

**Figure 3.**
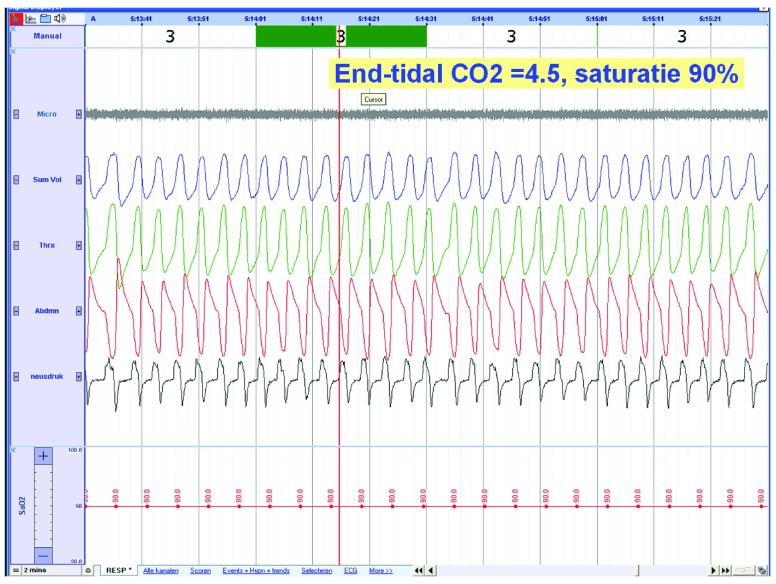
Polysomnographic tracing without ventilatory support showing paradoxical movements of thorax and abdomen (traces 3 and 4, respiratory inductive plethysmography). From the nasal pressure signal (trace 5) it can be seen that breathing movements follow the inspiration.

**Figure 4.**
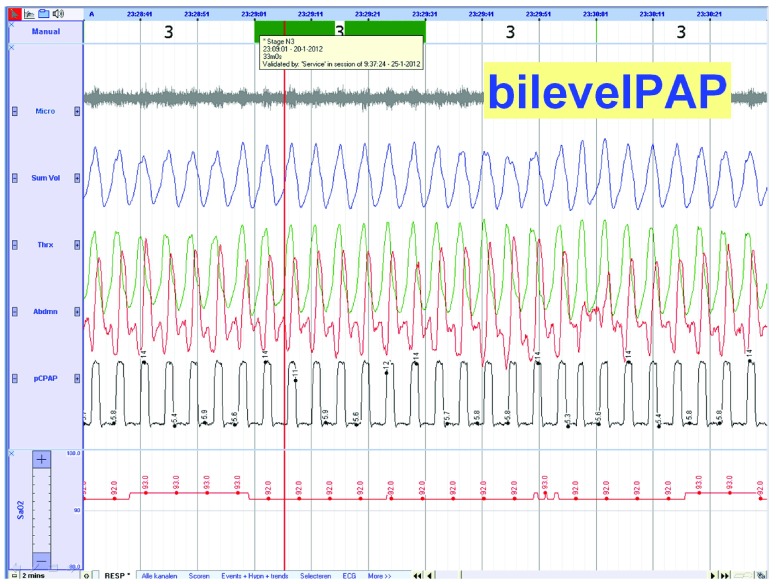
Polysomnography with ventilatory support (Bilevel PAP, IPAP=14, EPAP=6, see trace 5). Abdominal and thoracic movements are not completely in-phase because the ventilatory support is not triggered before there is inspiratory flow.

Ultrasonography showed lack of thickening of the diaphragm with inspiration indicating a non-functioning diaphragm. Polysomnography without ventilatory support showed periods of nocturnal desaturations together with out-of-phase thoracic and abdominal movement ( Figure 3 and Figure 4).

There was no clinical evidence of central neurological abnormalities. The electromyogram (EMG) of the diaphragm revealed a normal distal motor latency with normal CMAP-amplitude of phrenic nerve on both sides. Needle EMG revealed good recruitment without spontaneous muscle activity in the right hemi-diaphragm. Technically measurement of the hemi-diaphragm was less reproducible. In conclusion there was no evidence for traumatic phrenic nerve palsy.

An extended differential diagnosis of bilateral diaphragmatic paralysis is presented below in Table 1.

**Table 1.** Extended differential diagnosis of bilateral diaphragmatic paralysis.

Neurologic causes	Myopathic causes
Spinal cord transaction	Limb-girdle dystrophy
Multiple sclerosis	Hyperthyroidism
Amyotrophic lateral sclerosis	Malnutrition
Neuralgic amyotrophy	Acid maltase deficiency
Poliomyelitis	Connective tissues diseases
Guillan-Barre syndrome	Systemic lupus erythematosus
Phrenic nerve dysfunction	Dermatomyositis
Compression by tumor	Mixed connective tissues disease
Cardiac surgery cold injury	Amyloidosis
Blunt trauma	Idiopathic myopathy
Idiopathic phrenic neuropathy	
Post-viral phrenic neuropathy	
Radiation therapy	
Cervical chiropractic manipulation	

The diagnosis of Lyme disease was made on the basis of serological tests demonstrating recent infection with *B. burgdorferi*. The diagnosis of bilateral diaphragmatic weakness was made on the basis of fluoroscopy with a sniff test ( Figure 2) and ultrasonography of the diaphragm. The patient received oral doxycycline (200 mg q.d. for 4 weeks) and nocturnal support with NIV/BiPAP was started. Following therapy, our patient showed a dramatic improvement. He stopped using the nocturnal support of mechanical ventilation. He can now lie down in supine position without being orthopneic. The Epworth Sleepiness Scale (ESS) is obviously improved, and he has no other complaints. The repeated pulmonary function test showed improvement in the forced vital capacity (FVC) in supine position (from 31.5% to 65% predicted), however the difference between supine and upright position remain above the 20%. The pressure of the main inspiratory muscle is also improved in the follow-up. In the repeated polysomnography without ventilator support there was still dominant out-phase motion between abdomen and chest, which indicate persistent diaphragm dysfunction.

## Discussion

In our case the diagnosis was based on the clinical signs and symptoms, chest radiographs and serology indicating recent infection by *B. burgdorferi*. Our patient was not aware of a tick-bite one year before the initial presentation, but the numbness in the left side of his face and the skin erythema spontaneously resolved within a couple of weeks, put us on track. By definition, the nervous system involvement only occurs in the disseminated phase of the infection ^2^.

The symptoms of neurologic involvement may occur weeks to several months after tick bite and may be the first manifestation of Lyme disease ^1^. Neurological evaluation revealed no abnormalities in our patient. Although the facial nerve is the most commonly affected cranial nerve, the classic manifestations of acute neurologic abnormalities due to Lyme disease are meningitis, cranial neuropathy, and motor or sensory radiculoneuropathy. Each of these findings may also occur individually ^2^. Ventilatory support is very useful in acute respiratory impairment due to diaphragmatic weakness in a patient with Lyme disease.

In a case report of three patients with neuroborreliosis presenting with acute respiratory impairment, all patients presented respiratory failure associated with progressive nocturnal hypoventilation or prolonged central apnoea. Tracheostomy and prolonged periods of ventilatory support were necessary in all three cases. These cases emphasise that *Borrelia* infection should be considered in the differential diagnosis of unexplained respiratory failure ^7, 9, 10^. Bilateral diaphragmatic paralysis is a common cause of complete respiratory failure and the symptoms could be severe ^4, 5^.

In the literature only sporadic case reports comment on the respiratory failure due to Lyme disease ^3^.

In these cases, patients with respiratory failure caused by diaphragmatic paralysis due to Lyme disease were ventilated maximum for up to 2 months.

Our patient is clinically completely recovered, but he remains, despite improvement, respiratory insufficient according to the pulmonary function test, the polysomnography and the measurement of maximal inspiratory pressure. He shows a good acceptance of the nocturnal ventilatory support. We expect a successful recovery from the phrenic nerve palsy gradually in the next 2 to 3 years. In a group of 50 patients suffering of phrenic nerve palsy about 1/3 fully recovered, 1/3 recovered in 2–4 years and the rest showed no progress in recovering ^9^.

In conclusion, Lyme disease is an important differential diagnosis in patients with diaphragmatic paralysis. There can be an important delay between the tick bite and the development of symptoms, which has to be taken into account when dealing with these patients.

## Consent

Written informed consent for publication of clinical details and clinical images was obtained from the patient.
