# Supplementary material for: Case Report: Bilateral diaphragmatic dysfunction due to Borrelia Burgdorferi
Source: F1000Res. 2014 Oct 6;3:235. [Version 1] doi: 10.12688/f1000research.5375.1 (PMC4309166; doi:10.12688/f1000research.5375.1)
Supplement: Peer review history

# Peer review: Referee response for version 1

**DOI:** 10.5256/f1000research.5738.r7473

**Published:** 2015-01-27

**Authors:** W.N. Welvaart

**Affiliations:** 1 Department of Surgery, Riverland Hospital Tiel, Tiel, Netherlands

The authors report a patient with bilateral diaphragmatic dysfunction due to Borrelia.

I enjoyed reading this interesting and well written manuscript.

I think it would be nice to point out more details about the etiology of functional disorders of the diaphragm and acquired paralysis to explain why Lyme desease should be considered in the differential diagnosis of functional disorders of the diaphragm beside the mentioned extended differential diagnosis in table 1.

Despite my comments, I think that in its current form it can be accepted for indexing.

I have read this submission. I believe that I have an appropriate level of expertise to confirm that it is of an acceptable scientific standard.

# Peer review: Referee response for version 1

**DOI:** 10.5256/f1000research.5738.r7198

**Published:** 2015-01-05

**Authors:** Sezai Celik

**Affiliations:** 1 Thoracic Surgery Department, Siyami Ersek Cardiothoracic Training Hospital, Istanbul, Turkey

The authors report a patient with bilateral diaphragmatic paralysis due to Borrelia, which improved with antibiotherapy and non-invasive ventilation. The manuscript is quite well written and sufficiently well documented. The duration of  NIV/BiPAP should be clearly pointed out. 

İt can be accepted for indexing in its current form.

I have read this submission. I believe that I have an appropriate level of expertise to confirm that it is of an acceptable scientific standard.
